# Comparative Analysis of US Guidelines for the Management of Cutaneous Squamous Cell and Basal Cell Carcinoma

**DOI:** 10.1155/2024/3859066

**Published:** 2024-02-09

**Authors:** Amit Mittal, Bharat B. Mittal

**Affiliations:** ^1^Department of Dermatology, Mayo Clinic College of Medicine & Science, 200 First St SW, Rochester, MN 55905, USA; ^2^Department of Radiation Oncology, Northwestern University, 251 E Huron LC-178, Chicago, IL 60611, USA

## Abstract

**Background:**

This study presents a comparative analysis of recently published guidelines to manage cutaneous squamous cell carcinoma (cSCC) and cutaneous basal cell carcinoma (cBCC) within the United States (US).

**Methods:**

A PubMed database search was performed for the time period between June 1, 2016, and December 1, 2022. A comprehensive comparison was performed in the following clinical interest areas: staging and risk stratification, management of primary tumor and regional nodes with curative intent, and palliative treatment.

**Results:**

Guidelines from 3 organizations were analyzed: the American Academy of Dermatology (AAD), the National Comprehensive Cancer Network (NCCN), and the American Society for Radiation Oncology (ASTRO). The guidelines used different methodologies to grade evidence, making comparison difficult. There was agreement that surgery is the preferred treatment for curative cBCC and cSCC. For patients ineligible for surgery, there was a consensus to recommend definitive radiation. AAD and NCCN recommended consideration of other topical modalities in selected low-risk cBCC. Postoperative radiation therapy (PORT) was uniformly recommended in patients with positive margins that could not be cleared with surgery and in patients with nerve invasion. The definition and extent of nerve invasion varied. All guidelines recommended surgery as the primary treatment in patients with lymph node metastases in a curative setting. The criteria used for PORT varied; NCCN and ASTRO used lymph node size, number of nodes, and extracapsular extension for recommending PORT. Both NCCN and ASTRO recommend consideration of systemic treatment along with PORT in patients with extracapsular extension. Conclusion: US guidelines provide contemporary and complementary information on the management of cBCC and cSCC. There are opportunities for research, particularly in the areas of staging, indications for adjuvant treatment in curative settings, extent of nerve invasion and prognosis, and the role of systemic treatments in curative and palliative settings.

## 1. Introduction

Skin cancer is the most common malignancy in the United States (US). The most common skin malignancies are cutaneous basal cell carcinoma (cBCC) and cutaneous squamous cell carcinoma (cSCC) [1]. Although they have excellent local control and survival rates, there is wide variation in the management of these tumors, with few randomized trials comparing the different treatment modalities [2–4]. To provide recommendations based on the best available evidence and expert opinion, several international and US organizations have published guidelines to manage these tumors [5–12].

In the US, the National Comprehensive Cancer Network (NCCN) was the first organization to publish consensus-based guidelines in 1999 for the management of skin malignancies. These were recently updated in 2022 [12]. In 2018, the American Academy of Dermatology (AAD) published their guidelines to manage cBCC and cSCC [9, 10]. In 2019, the American Society for Radiation Oncology (ASTRO) published guidelines with an emphasis on indications for radiation therapy [11].

A comparative analysis of International and US guidelines in patients with high risk and advanced cSCC has been published [13], but no such comparison has been performed amongst US guidelines. The aim of this review is to compare recently published US guidelines for the management of cBCC and cSCC and point out future opportunities for research.

## 2. Materials and Methods

We developed search strategies in PubMed that combined and incorporated medical subject headings and text words. The first search incorporated terms for the NCCN, squamous cell carcinoma, and basal cell carcinoma. The second search combined terms for guidelines, squamous cell carcinoma, basal cell carcinoma, and diagnosis and management. The search terms included cutaneous, skin, basal, squamous, cancer, carcinoma, and guidelines. We limited the search to studies published between June 1, 2016, and December 1, 2022.

To compare various US guidelines, the following areas of clinical interest were reviewed and compared: methodology of guideline development, staging and risk stratification, management of primary tumors and regional nodes with curative intent, and palliative treatment.

## 3. Results

**Figure 1:**
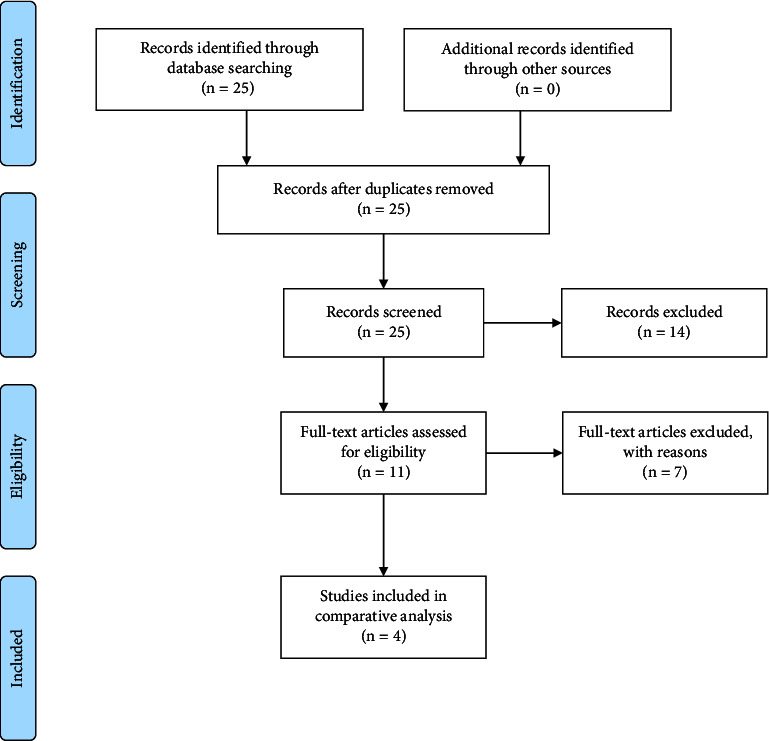
Preferred reporting items for systematic review and meta-analysis (PRISMA) flowchart used for identification of articles.

A total of 25 published articles were identified relevant to our study (Figure 1). Following abstract review, 11 articles underwent full review. Of these, 7 articles were excluded; 1 was Spanish guidelines for the management of cBCC [14], 1 was Swiss guidelines for the management of cBCC [15], 2 were British guidelines for the management of adults with cSCC and cBCC [16, 17], 1 was on consensus management of actinic keratosis [18], 1 described guidelines for the follow-up of patients treated with hedgehog inhibitors [19], and 1 presented US preventive service task force recommendation for screening skin cancer [20]. The remaining 4 US guidelines, 2 from the AAD, 1 from the NCCN, and 1 from the ASTRO, form the basis of this report [9–12].

### 3.1. Panel Expertise

The specialties represented on the expert panel could potentially bias treatment recommendations in the absence of high-level evidence.  Table 1 lists the specialties represented on the AAD, NCCN, and ASTRO panels.

### 3.2. Methodology of Guideline Development

US guidelines used different methodologies to evaluate available evidence and give recommendations, making interguideline comparisons difficult. The AAD evaluated the evidence using a unified system called the Strength of Recommendation Taxonomy (SORT). Treatment recommendations were developed based upon the quality of evidence and expert opinion (Table 2). The ASTRO guidelines were developed in accordance with the National Academy of Medicine standards. The available evidence for key questions was assessed using the Population, Intervention, Comparator, Outcome, Timing, Setting (PICOTS) framework. The Delphi approach was used to develop consensus (Table 2). NCCN guidelines are a statement of consensus of the panel members regarding their views of currently accepted approaches to cancer treatment (Table 2).

### 3.3. Staging and Risk Stratification

Several staging and risk stratification systems were discussed, including NCCN risk stratification [12], the American Joint Committee on Cancer (AJCC) Staging Manual-8^th^ edition [21], and Brigham and Women's Hospital (BWH) tumor classification [22, 23]. AAD and NCCN used low- and high-risk criteria as defined by NCCN to give their recommendations. In addition, NCCN categorized the cSCC high-risk group into high and very high-risk groups; the high-risk group has elevated risk of local recurrence, and the very high-risk group has elevated risk of local recurrence and metastasis [12]. NCCN used AJCC criteria for the management of neck nodes. ASTRO advocated the use of AJCC staging for their recommendations. There was moderate overlap between the guidelines.

### 3.4. Management of Primary Tumors with Curative Intent

There is general agreement that due to the paucity of data from well-designed randomized trials, most of the recommendations are based upon observational studies, expert opinion, and consensus of the panel members. There is consensus that the recommended treatments should take into consideration the best tumor control, cosmesis, function preservation, and patients' expectations.

#### 3.4.1. Surgical Management

All of the guidelines advocated surgery as the preferred treatment (Table 3). Details of surgical procedures were beyond the scope of the ASTRO guidelines. The indications for standard excision with “bread loaf” histopathologic sectioning with a 4–6 mm margin were the same in the AAD and NCCN guidelines, though the SOR varied from A to C in AAD, while the category of evidence and consensus was the same throughout the NCCN guidelines. The selection of patients for Mohs surgery and curettage and electrodessication (C & E) was the same between AAD and NCCN guidelines. The only difference was that in the NCCN guidelines, other forms of peripheral and deep enface margin assessment (PDEMA) were recommended along with Mohs for the treatment of high-risk tumors. C & E was recommended in both AAD and NCCN guidelines for low-risk tumors, excluding tumors of terminal hair-bearing areas.

#### 3.4.2. Radiotherapeutic Management

- Definitive Treatment: there was uniformity in all guidelines in recommending definitive radiation to patients who decline or cannot undergo surgery (Table 4), though the SOR was strongest in the ASTRO guidelines. Definitive radiation was conditionally recommended in ASTRO guidelines to preserve cosmesis and function.
- Postoperative Radiation Therapy (PORT): the indications for PORT varied between guidelines (Table 4). There was consensus to use PORT in patients with nerve invasion, though the definition and extent of nerve invasion and SOR for PORT varied significantly between guidelines. PORT was recommended uniformly in patients with positive margins only if the margins cannot be corrected with surgery. ASTRO guidelines strongly recommended PORT in patients with recurrent disease, T3 and T4 tumors, and desmoplastic and infiltrative tumors in the setting of chronic immunosuppression. NCCN guidelines also suggested considering PORT in high-risk and very high-risk patients, while AAD guidelines did not explicitly comment on additional risk factors as an indication for PORT.

#### 3.4.3. Imiquimod, 5-Fluorouracil, Cryosurgery, and Photodynamic Therapy

Amongst the AAD and NCCN guidelines, there was consensus that these modalities have no role in the definitive treatment of SCC, except that AAD recommended cryosurgery for the treatment of low-risk SCC only if other more effective therapies are contraindicated (Table 5). Discussion of these modalities was beyond the scope of ASTRO guidelines.

For cBCC, there was agreement between the AAD and NCCN guidelines that these modalities can be used only for low-risk superficial cBCC where other more effective treatments are contraindicated. In the AAD guidelines, the level of evidence and SOR was high due to available data from randomized trials comparing a variety of treatment modalities.

### 3.5. Management of Metastatic Regional Nodes with Curative Intent

There was agreement between the AAD and NCCN guidelines in managing cBCC with lymph node metastases, both recommended surgery ± PORT and hedgehog inhibitors as indicated (Table 6). For cSCC, surgery ± PORT was recommended by both the AAD and NCCN in patients with regional lymph node metastases. However, in the NCCN guidelines, the use of PORT was dependent on the size and the number of nodes and presence of extracapsular extension (ECE). Any patients with a lymph node >3 cm, ≥2 regional nodes, and ECE were recommended to have PORT. Patients with ECE were advised to consider systemic therapy in addition to PORT. In the ASTRO guidelines, cBCC and cSCC were addressed together. There were similarities between the NCCN and ASTRO guidelines for PORT recommendations to patients with nodes >3 cm and/or ECE. ASTRO guidelines also conditionally recommended elective nodal radiation to patients with SCC at a high risk of regional nodal metastases.

### 3.6. Management of Distant Metastases and Advanced Disease with Palliative Intent

All guidelines encouraged clinical trials, multidisciplinary consultation, and management with supportive care. There was uniformity in recommending various combinations of surgery, radiation, platinum-based chemotherapy, epidermal growth factor receptor inhibitors, and immunomodulators, depending on the clinical scenario. All guidelines recommended hedgehog inhibitors in patients with cBCC with distant metastases.

## 4. Discussion

We present a comparative analysis of recently published US guidelines to manage cBCC and cSCC. The differences in methodology, grading for quality of evidence, staging, and risk stratification make comparison between guidelines challenging. There was agreement between guidelines that, due to limited data from randomized trials, most of the recommendations are based upon retrospective studies, expert opinions, and consensus of the panel members. The expertise represented on the panels could potentially bias recommendations, particularly in the absence of high-level evidence.

In 2013, Brigham and Women's Hospital's (BWH) tumor classification system was proposed and later validated [22, 23] for the management of cSCC due to the poor prognostic value of AJCC staging. However, BWH classification lacks the inclusion of lymph node and distant metastases, which are included in the AJCC classification. NCCN risk stratification has been widely adopted and was used in both the AAD and NCCN guidelines. Currently, there is no consensus on which staging or risk stratification system is optimum in managing and predicting the outcome for cSCC and it is an area of active research.

Heppt et al. [13] recently published a comparative analysis of guidelines for managing high-risk and advanced cSCC from the US, Canada, the United Kingdom, the European Union, and Italy. The authors noted that there was consensus on several treatment strategies; however, there were differences in the management recommendations, SOR related to surgical margins, indications for sentinel node biopsy, use of PORT, and the treatment of metastatic disease.

Our analysis mirrors the observations made by Heppt et al. [13]. We observed significant points of agreement between guidelines, but there were differences also. There was general agreement on the use of surgery, surgical margins of 4–6 mm, and surgery as the preferred curative treatment for primary sites. The AAD recommended Mohs to treat high-risk tumors, while NCCN categorized high-risk into high and very high-risk groups and added other forms of peripheral and deep enface margin assessment (PDEMA) to Mohs to manage these tumors. The randomized data to support use of MMS are from van Loo et al., who reported a 5-year cumulative probability of recurrence of 12.2% vs. 4.4% in patients treated with surgical excision vs. MMS, respectively [3]. Details of reconstruction of surgical defect, though beyond the scope of this manuscript, are important in order to achieve an optimum treatment outcome. Guidelines for reconstruction after resection of skin cancer are published by Chen et al. [24].

The role of radiation was discussed more comprehensively in ASTRO and NCCN guidelines. There was agreement between guidelines to use definitive radiation if surgery is not possible or advisable. Effectiveness of radiation as the primary management is based largely on observation studies [25, 26]. The indications of PORT for primary management varied between guidelines. There was general agreement to use PORT in patients with perineural invasion (PNI) and positive margins only if the margin cannot be corrected with additional surgery. In addition, ASTRO guidelines strongly recommend PORT in patients with tumors invading bones or tumors >4 cm in the largest dimension. The definition and extent of nerve invasion for PORT varied from gross perineural spread that is clinically or radiographically apparent (ASTRO) to PNI without further characterization (AAD) and extensive PNI spread to large or named nerves (>0.1 mm in diameter; NCCN). The SOR for PORT was strong in the ASTRO guidelines. Retrospective studies have shown that local control is much higher in patients with incidental PNI compared to clinical PNI [27], minimal to moderate PNI compared to central or macroscopic PNI [28], and microscopic focal PNI (involvement of 1-2 nerves <0.1 mm diameter) compared to macroscopic extensive PNI (involvement of >2 nerves) [29]. There was no universal agreement on the risk category based on the extent of PNI. This is a potential area for future research. In order to reduce variations among clinicians, a group of experts published international radiation treatment contouring guidelines in the postoperative setting to treat patients with complex cSCC of the head and neck area [30].

In the AAD and NCCN guidelines, treatment modalities such as imiquimod, 5-fluorouracil, cryosurgery, and photodynamic therapy are contraindicated for cSCC but can be used in selected low-risk superficial cBCC if other treatment modalities are contraindicated. The level of evidence and SOR was high in AAD guidelines due to available data from randomized trials [31–33].

The risk for regional nodal metastases is uncommon but higher in immunocompromised patients. Both ASTRO and NCCN guidelines were aligned and used the AJCC Staging Manual—8^th^ Edition criteria to recommend PORT depending on the nodal size, number of positive nodes, and ECE. AAD guidelines suggest considering PORT in patients with neck metastases without further clarification. All guidelines suggest considering adjuvant chemotherapy or participation in a clinical trial. However, in a phase III trial of high-risk cSCC of the head and neck, there was no benefit of adding chemotherapy to radiation treatment [4]. Elective treatment of regional nodes is an area of controversy. Wilkie et al. [34] published a contemporary perspective in the management of regional nodal basin in patients with cSCC. The management of regional nodes is another potential area for research.

For patients treated with palliative intent, clinical trial participation, and multidisciplinary consultation was uniformly recommended. There was general agreement on the use of hedgehog inhibitors in cBCC. Use of surgery, radiation, platinum-based chemotherapy, epidermal growth factor receptor inhibitors, and immunomodulators were recommended depending on the clinical scenario.

Recent advances in the understanding of cancer biology and the mechanism by which cancer creates an immunologically privileged microenvironment for the malignant cells to survive afford an opportunity for ongoing and future research. cSCC is an immunogenic tumor with a high mutational burden [35, 36]. Checkpoint inhibitors such as pembrolizumab and cemiplimab have shown clinically meaningful activity against recurrent or metastatic and unresectable cSCC [37–39]. Recently published results from a phase II trial confirmed that neoadjuvant cemiplimab was associated with a pathological complete response in a high percentage of patients with resectable cSCC [40]. Integration of 40-gene expression profiling (40-GEP) in the management of cSCC and advances in artificial intelligence and data science will create additional opportunities in the diagnosis and management of cBCC and cSCC [41–45].

## 5. Conclusion

US guidelines provide contemporary and complementary information on the management of cBCC and cSCC. There are significant points of agreement and few disagreements between the guidelines. In spite of different criteria used for grading the evidence and potential bias introduced by experts on the panels, the guidelines are useful in clinical practice by reducing variability and maintaining quality care. The discordance in treatment recommendations can be harmonized by creating a national task force of stakeholders. Due to limited data from randomized trials, there are significant opportunities for future research.

## Figures and Tables

**Table 1:** Specialties represented on expert panels.

AAD	NCCN	ASTRO
Dermatology Plastic and reconstructive surgery Head and neck surgery Radiation oncology Pathology Family medicine Internal medicine	Dermatology Plastic and reconstructive surgery Surgical oncology Radiation oncology Pathology Radiology Medical oncology Otolaryngology Internal medicine Patient advocacy	Radiation oncology Surgical oncology Medical oncology

AAD: American Academy of Dermatology (Reference #[9, 11]), NCCN: National Comprehensive Cancer Network (Reference #[13]), and ASTRO: American Society of Radiation Oncology (Reference #[12]).

**Table 2:** Quality of evidence.

AAD		ASTRO	
Grade	Definition	Grade	Definition
I	Good-quality patient-oriented evidence (i.e., evidence-measuring outcomes that matter to patients: morbidity, mortality, symptom improvement, cost reduction, and quality of life)	High	(i) 2 or more well-conducted and highly generalizable RCTs or meta-analyses of such trials
II	Limited-quality patient-oriented evidence	Moderate	(i) 1 well-conducted and highly generalizable RCT or a meta-analysis of such trials OR
			(ii) 2 or more RCTs with some weaknesses of procedure or generalizability OR
			(iii) 2 or more strong observational studies with consistent findings
III	Other evidence, including consensus guidelines, opinion, case studies, or disease-oriented evidence (i.e., evidence measuring intermediate, physiologic, or surrogate end points that may or may not reflect improvements in patient outcomes)	Low	(i) 1 RCT with some weaknesses of procedure or generalizability OR
			(ii) 1 or more RCTs with serious deficiencies of procedure or generalizability or extremely small sample sizes OR
			(iii) 2 or more observational studies with inconsistent findings, small sample sizes, or other problems that potentially confound interpretation of data
Expert opinion^*∗*^		Expert opinion^*∗*^	

Strength of recommendation			
AAD		ASTRO	
Ranking	Definition	Strength	Definition

A	Recommendation based on consistent and good-quality patient-oriented evidence	Strong	(i) Benefits clearly outweigh risks and burden, or risks and burden clearly outweigh benefits
			(ii) All or almost all informed people would make the recommended choice
B	Recommendation based on inconsistent or limited-quality patient-oriented evidence	Conditional	(i) Benefits are finely balanced with risks and burden or appreciable uncertainty exists about the magnitude of benefits and risks
C	Recommendation based on consensus, opinion, case studies, or disease-oriented evidence		(ii) Most informed people would choose the recommended course of action, but a substantial number would not
			(iii) A shared decision-making approach regarding patient values and preferences is particularly important

NCCN guidelines version 2.2022			
NCCN categories of evidence and consensus			

Category 1	Based upon high-level evidence, there is uniform NCCN consensus that the intervention is appropriate		
Category 2A	Based upon lower-level evidence, there is uniform NCCN consensus that the intervention is appropriate		
Category 2B	Based upon lower-level evidence, there is NCCN consensus that the intervention is appropriate		
Category 3	Based upon any level of evidence, there is major NCCN disagreement that the intervention is appropriate		

(Adapted from References [9–11]). AAD = American Association of Dermatology, ASTRO = American Society of Radiation Oncology, RCT = randomized controlled trial, and ^*∗*^expert opinion of panel members used due to the absence of or limitation in published evidence-based data.

**Table 3:** Management of primary tumor with curative intent: surgical management.

Treatment	AAD			NCCN		ASTRO		
	Narrative	LOE	SOR	Narrative	COE	Narrative	QOE	SOR
Standard excision						Details of surgical management outside the scope of ASTRO guidelines		
SCC								
Low risk	4–6 mm margin to depth of subcutaneous adipose tissue	II	B	4–6 mm margin and postoperative margin assessment	2A			
High risk	Select tumors	II	B	Select tumors. Wider surgical margin and postoperative margin assessment	2A			
Very-high risk	Not defined	II	B	Select tumors. Wider surgical margin and postoperative margin assessment	2A			
BCC								
Low risk	4 mm margin	I	A	4 mm margin and postoperative margin assessment	2A			
High risk	Select tumors	II, III	C	Select tumors. Wider surgical margin with postoperative margin assessment	2A			
Mohs/PDEMA								
SCC	High-risk tumors	II, III	B	High/very high-risk tumors	2A			
BCC	High-risk tumor	I, II	A	High-risk tumors	2A			
C & E								
SCC	Low-risk tumors. Exclude lesions of terminal hair-bearing areas	II, III	B	Low-risk tumors. Exclude terminal hair-bearing skin lesions	2A			
BCC	Low-risk tumors. Exclude tumors of terminal hair-bearing areas	I, II	B	Low-risk tumors. Exclude terminal hair-bearing skin lesions	2A			

AAD: American Academy of Dermatology (Reference #[9, 11]), NCCN: National Comprehensive Cancer Network (Reference #[13]), ASTRO: American Society of Radiation Oncology (Reference #[12]), LOE: level of evidence, QOE: quality of evidence, SOR: strength of recommendation, COE: category of evidence, C & E: curettage and electrodessication,and PDEMA: peripheral and deep enface margin assessment.

**Table 4:** Management of primary tumor with curative intent: radiation therapy.

Treatment	AAD			NCCN		ASTRO		
	Narrative	LOE	SOR	Narrative	COE	Narrative	QOE	SOR
Radiation therapy								
Definitive	Indicated if surgery not feasible, contraindicated, or not preferred by the patient	II, III	B	Recommended for nonsurgical candidates	2A	Indicated if the patient cannot undergo or declines surgery	Moderate	Strong
						If surgery will compromise cosmesis or function	Moderate	Conditional
						Genetic conditions predisposing to increased radiosensitivity	Expert opinion	Conditional
PORT								
Both BCC & SCC	Consider for perineural invasion	II, III	B	Extensive perineural spread. Large or named nerve (>0.1 mm diameter)	2A	Gross perineural spread, clinically or radiologically	Moderate	Strong
cSCC	Not explicitly stated			Positive margins cannot be corrected with surgery	2A	Close or positive margin cannot be corrected with surgery	Low	Strong
				Consider in high/very high-risk NCCN features	2A	Setting of recurrent disease following prior margin negative resection	Moderate	Strong
					2A	Recommended for T3 and T4 tumors	Moderate	Strong
					2A	Desmoplastic or infiltrative tumors in the setting of chronic immunosuppression	Moderate	Strong
cBCC	Not explicitly stated			Positive margin where reexcision is not feasible		Close or positive margin where reexcision is not feasible	Low	Conditional
						Tumor recurrence after a prior margin negative resection	Low	Conditional
				Consider adjuvant radiation in patients with residual disease where surgery is not feasible		Tumor infiltrating bone or muscle	Low	Conditional

AAD: American Academy of Dermatology (Reference #[9, 11]), NCCN: National Comprehensive Cancer Network (Reference #[13]), ASTRO: American Society of Radiation Oncology (Reference #[12]), LOE: level of evidence, QOE: quality of evidence, SOR: strength of recommendation, COE: category of evidence, and PORT: postoperative radiation therapy.

**Table 5:** Management of primary tumor with curative intent: nonsurgical treatments.

Treatment	AAD			NCCN		ASTRO		
	Narrative	LOE	SOR	Narrative	COE	Narrative	QOE	SOR
SCC						Beyond the scope of ASTRO guidelines		
Imiquimod	Not recommended	III	C	Not recommended				
5-Fluorouracil	Not recommended	III	C	Not recommended				
Photodynamic therapy	Not recommended	II	B	Not recommended				
Laser therapy	Not recommended	III	C	Not recommended				
Cryosurgery	Only for low-risk tumors where other effective treatments are contraindicated	II	B	Not recommended				
BCC								
Imiquimod	Reserved for low-risk tumors where other definitive treatments are contraindicated	I	A	Not routinely recommended but consider for low-risk BCC where other more effective treatments are contraindicated				
5-Fluorouracil	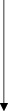	I, II	B	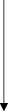				
Photodynamic therapy		I, II	A					
Cryosurgery		I	A					

AAD: American Academy of Dermatology (Reference #[9, 11]), NCCN: National Comprehensive Cancer Network (Reference #[13]), ASTRO: American Society of Radiation Oncology (Reference #[12]), LOE: level of evidence, QOE: quality of evidence, SOR: strength of recommendation, and COE: category of evidence.

**Table 6:** Management of metastatic regional nodes with curative intent.

Treatment	AAD			NCCN		ASTRO		
	Narrative	LOE	SOR	Narrative	COE	Narrative	QOE	SOR
BCC	Surgery ± PORT	I, II	A	Surgery ± PORT		BCC and SCC inclusive	Moderate	Strong
	Hedgehog inhibitors			Hedgehog inhibitors		Surgery + PORT for patients with a node >3 cm, multiple nodes, and/or with ECE		
	(i) vismodegib			(i) Vismodegib	2A			
	(ii) Sonidegib			(ii) Sonidegib	2B			
				(iii) Cemiplimab rwlc	2A			

SCC	Surgery ± PORT	II	B	Surgery ± PORT	2A	Elective nodal radiation suggested in SCC with a high risk of regional metastases	Conditional	Expert opinion
				(i) ≤3 cm single node no ECE: ±PORT				
				(ii) ≥2 positive node, 1 node >3 cm, no ECE: PORT yes				
				Surgery + PORT ± systemic therapy	2A			
				(i) Any node with ECE				

AAD: American Academy of Dermatology (Reference #[9, 11]), NCCN: National Comprehensive Cancer Network (Reference #[13]), ASTRO: American Society of Radiation Oncology (Reference #[12]), LOE: level of evidence, QOE: quality of evidence, SOR: strength of recommendation, COE: category of evidence, PORT: postoperative radiation therapy, and ECE: extracapsular extension.

## Data Availability

The data that support the findings of this study will be shared upon reasonable request to the corresponding author.
